# Does sex matter in the cheetah? Insights into the skeletal muscle of the fastest land animal

**DOI:** 10.1242/jeb.247284

**Published:** 2024-08-01

**Authors:** Tertius A. Kohn, Samantha Knobel, Byron Donaldson, Kathryn M. van Boom, Dee M. Blackhurst, James M. Peart, Jørgen Jensen, Adrian S. W. Tordiffe

**Affiliations:** ^1^ Department of Medical Biosciences, Faculty of Natural Sciences, University of the Western Cape, Cape Town, 7530, South Africa; ^2^ Department of Human Biology, University of Cape Town, Cape Town, 7925, South Africa; ^3^ Centre for Veterinary Wildlife Research and Department of Paraclinical Sciences, Faculty of Veterinary Science, University of Pretoria, Pretoria, 0110, South Africa; ^4^ Division of Chemical Pathology, Department of Pathology, University of Cape Town, Cape Town, 7925, South Africa; ^5^ Department of Physical Performance, Norwegian School of Sport Sciences, 0863 Oslo, Norway

**Keywords:** Fibre type, Enzymes, Metabolism, *Acinonyx jubatus*

## Abstract

The cheetah is considered the fastest land animal, but studies on their skeletal muscle properties are scarce. Vastus lateralis biopsies, obtained from male and female cheetahs as well as humans, were analysed and compared for fibre type and size, and metabolism. Overall, cheetah muscle had predominantly type IIX fibres, which was confirmed by the myosin heavy chain isoform content (mean±s.d. type I: 17±8%, type IIA: 21±6%, type IIX: 62±12%), whereas human muscle contained predominantly type I and IIA fibres (type I: 49±14%, type IIA: 43±8%, type IIX: 7±7%). Cheetahs had smaller fibres than humans, with larger fibres in the males compared with their female counterparts. Citrate synthase (16±6 versus 28±7 µmol min^−1^ g^−1^ protein, *P*<0.05) and 3-hydroxyacyl co-enzyme A dehydrogenase (30±11 versus 47±15 µmol min^−1^ g^−1^ protein, *P*<0.05) activities were lower in cheetahs than in humans, whereas lactate dehydrogenase activity was 6 times higher in cheetahs (2159±827 versus 382±161 µmol min^−1^ g^−1^ protein, *P*<0.001). The activities of creatine kinase (4765±1828 versus 6485±1298, *P*<0.05 µmol min^−1^ g^−1^ protein) and phosphorylase (111±29 versus 216±92 µmol min^−1^ g^−1^ protein) were higher in humans, irrespective of the higher type IIX fibres in cheetahs. Superoxide dismutase and catalase, markers of antioxidant capacity, were higher in humans, but overall antioxidant capacity was higher in cheetahs. To conclude, fibre type, fibre size and metabolism differ between cheetahs and humans, with limited differences between the sexes.

## Teaser

**Summary:** The skeletal muscle properties of male and female cheetahs are predominantly fast twitch orientated and lacks oxidative capacity due to low mitochondrial enzyme activities compared to humans.

## INTRODUCTION

The cheetah (*Acinonyx jubatus*) is considered the fastest land animal in the world, reportedly reaching speeds of 120 km h^−1^, with a recorded maximum speed of 106 km h^−1^ (Sharp, 1997). Although anatomy, locomotory dynamics and some skeletal muscle properties have been investigated, the physiology behind their high running speed remains inadequate. Humans have some of the best endurance (depending on training status), but could be seen as one of the slowest species in comparison to cheetahs, with the latter considered as having poor resistance to fatigue (Curry et al., 2012; Hetem et al., 2013; Kohn et al., 2011a). In humans, muscle strength and resistance to fatigue have been attributed to specific muscle fibre characteristics, with the latter resulting from adaptations that occur directly from the type of muscle activity (e.g. resistance or endurance training) (Gollnick et al., 1973, 1972). Comparative studies on wild animal muscle suggest that their muscle fibre type and metabolism are primarily determined by genetic factors that translate to their respective physical attributes (Kohn, 2014; Kohn et al., 2011a).

Skeletal muscle is a dynamic tissue that generates force to produce, amongst its many functions, locomotion in animals. A muscle comprises elongated multinucleated cells (i.e. fibres) with the smallest contractile unit being the sarcomere (Frontera and Ochala, 2015). In larger mammals, there are generally three major muscle fibre types, which are determined by the specific myosin heavy chain (MHC) isoform each fibre expresses, encoded by unique genes. These three isoforms are MHC I, MHC IIA and MHC IIX, that give rise to type I, IIA and IIX fibres, respectively, each with varying characteristics. Each of these MHC isoforms contain a unique myosin ATPase enzyme embedded within the myosin head, with each having a different ATP hydrolysis rate (Bottinelli and Reggiani, 2000; Frontera and Ochala, 2015; Kohn et al., 2011a). These differences give rise to slow (MHC I), medium (MHC IIA) and fast (MHC IIX) hydrolysis rates and are partially responsible for the contraction speed of the fibres. A fourth isoform, namely MHC IIB, frequently expressed in most rodent species, has been identified in one muscle group of the cheetah, and specialised muscles from humans (such as the eye) (Hyatt et al., 2010; Kohn and Myburgh, 2007).

The three fibre types also rely on metabolic processes that must supply ATP at a rate that equals ATP demand by the myosin heads for muscle contraction and relaxation to occur. Type I fibres are highly oxidative, contain large mitochondrial numbers, and produce ATP primarily from oxidative phosphorylation via the glycolytic–Krebs cycle and β-oxidation of carbohydrate and fat, respectively (Hargreaves and Spriet, 2020; Kohn et al., 2011c). Type IIX fibres, requiring ATP at a much higher rate than type I and IIA fibres, rely more on anaerobic glycolysis of glucose to lactate and phosphocreatine stores, as this fibre type conventionally harbours low mitochondrial numbers. However, data have shown that certain species, such as the dog and various antelope species, contain oxidative type IIX fibres (Acevedo and Rivero, 2006; Curry et al., 2012; van Boom et al., 2023). Type IIA fibres, which are fast twitch oxidative fibres, with mitochondrial numbers somewhat similar to those of type I fibres, can derive ATP from both oxidative and anaerobic pathways, making them an efficient fibre when speed and power are required (Pette, 2002; van Boom et al., 2023). It is also well known that muscle fibre cross-sectional area can also contribute to the amount of force a fibre can produce (Bottinelli and Reggiani, 2000). In all, structure, metabolism and fibre type contribute significantly towards muscle performance and may help explain superior performance (i.e. strength and running speed) in various species, including humans.

The speed and poor fatigue resistance observed in cheetahs have been attributed to their muscle fibre type and metabolism. Human muscles usually comprise a higher proportion of slow twitch oxidative (type I) and oxidative fast twitch (type IIA) fibres, whereas cheetah muscles comprise predominantly type IIX fibres (Goto et al., 2013; Kohn et al., 2007a,b; Williams et al., 1997). Apart from physical activity, which plays a significant role in muscle fibre type adaptations, it is well known that the sex of an animal can significantly contribute to muscle dimorphism as a result of the influence of genetics and hormones. Interestingly, muscle fibre type of the same muscle is mostly similar in proportion between men and women matched for physical activity. However, the size of the muscle fibres, where men usually harbour larger fibres than women, explains the disparity in strength between the sexes (Esbjornsson et al., 2021; Simoneau and Bouchard, 1989). Indeed, other factors that directly affect muscle function, e.g. genetics, behaviour, lifestyle (sedentary versus active lifestyle, endurance versus strength training), can obscure the influence that sex has on muscle function in humans (Haizlip et al., 2015; O'Reilly et al., 2021). In contrast, the influence of sex on the muscle characteristics of cheetahs is currently unknown.

While several researchers have evaluated the anatomical characteristics that enable cheetahs to accelerate to such phenomenal speeds (Goto et al., 2013; Hudson et al., 2011a,b), and technological advances have facilitated a better understanding of their locomotion and biomechanics (Shield et al., 2023; Wilson et al., 2013), research on cheetah muscle characteristics has had several limitations, including animals that had to be euthanised because of illness, opportunistic sampling, and small sample sizes (Goto et al., 2013; Hyatt et al., 2010; West et al., 2013; Williams et al., 1997). As health, age and sex of an animal play a significant role in muscle morphology and function, there remain unanswered questions regarding the muscle characteristics of the cheetah as a result of these limitations in the previous studies. Nevertheless, the available data suggest that cheetahs possess predominantly fast twitch type IIX fibres (Goto et al., 2013; Hyatt et al., 2010; Williams et al., 1997), have high anaerobic capacity (via the conversion of pyruvate to lactate) and poor oxidative capacity, observed from low mitochondrial numbers and concomitant markers of these pathways, especially in citrate synthase (CS) activity (Williams et al., 1997).

As cheetahs are considered vulnerable and endangered, many of these animals are protected through conservation programmes in captive or semi-captive camps, which pose significant lifestyle challenges to these animals such as in diet and a lack of physical activity (van Boom et al., 2024). Thus, a better understanding of the muscle physiology of healthy male and female wild cheetahs will be important benchmarks for the future development of better conservation programmes (Haizlip et al., 2015).

The present study used a mixed comparative model approach that included cheetah and human recreationally active individuals from both sexes to better understand muscle function and exercise performance. To elucidate the muscle physiology of the cheetah, the present study aimed to investigate the muscle fibre type and fibre sizes, as well as the energy-providing and antioxidant pathways of the vastus lateralis (VL) muscle of adult cheetahs and compare the findings with those of the VL from recreationally active adult humans. An additional aim was to determine whether sex plays a definitive role in these properties.

## MATERIALS AND METHODS

### Ethical approval and permits

Ethical approval for this study was obtained from the National Zoological Gardens Research Ethics and Scientific Committee (Ref. P14/12) and the University of Cape Town Animal Research Ethics Committee (Ref. 15/028). Threatened or Protected Species (TOPS; Ref. 05238) and CITES (Ref. 0042838 and 147527) permits for animals were obtained as well as the required Section 20 research permit of the Republic of South Africa to comply with the Animal Diseases Act of 1984 (Ref. 12/11/1/1/18).

For the human participants, ethical approval was obtained from the University of Cape Town Human Research Ethics Committee (Ref. 556/2012) for which all participants provided written informed consent.

### Animals and sampling

**Fig. 1.**
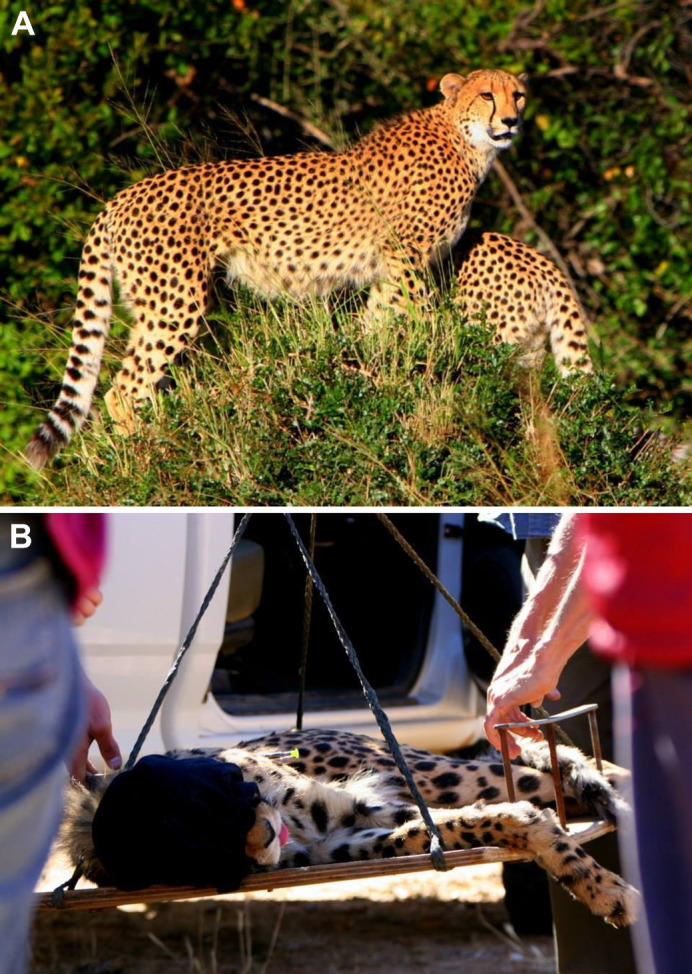
**Photographs of cheetahs at the AfriCat Foundation.** (A) Two male cheetahs. (B) Darting and immobilisation of a cheetah and transportation to the field laboratory. Photo credit: A.S.W.T.

Thirty-four cheetahs (20 males and 14 females) from the AfriCat Foundation on the Okonjima Private Nature Reserve, Namibia, were included in this study (Fig. 1). These cheetahs had been deemed unfit to return to the wild for various reasons (e.g. orphaned cubs raised in captivity, human habituation and sterilisation of females, which has been shown to compromise their survival in the wild) and remained in semi-captivity under the care of the AfriCat Care Centre (Kirberger and Tordiffe, 2016). Here, cheetahs are provided with approximately 10,000 m^2^ roaming area per captive carnivore and fed a controlled diet of 1.2 kg of donkey meat, 6 days a week (fasted on day 7). All cheetahs used in the study fell within the age range of 2 to 13 years. Additionally, all diseased, disabled or pregnant individuals were excluded from the study.

VL muscle biopsy samples (∼300 mg per animal) were obtained using the open biopsy technique during an annual health check at the AfriCat Foundation, conducted by a wildlife veterinarian (June and July 2014). Briefly, fasted cheetahs were immobilised using a dart gun with a combination of tiletamine and zolazepam (Zoletil, Virbac, Halfway House, South Africa) (0.9−1.4 mg kg^−1^) and medetomidine (Domitor, Pfizer Animal Health, Sandton, South Africa) (0.03–0.045 mg kg^−1^) (Fig. 1B). For anaesthetic maintenance, isoflurane in oxygen (Isofor, Safeline Pharmaceuticals, Johannesburg, South Africa) was used. After all procedures were completed, the medetomidine was reversed with its antagonist, atipamezole (Antisedan, Pfizer Animal Health) (0.08–0.12 mg kg^−1^) (Kirberger and Tordiffe, 2016).

During the procedures, the superficial mid-portion of the VL was used as the standard anatomical location for all biopsies. The midway distance between the hip and knee joint was used as the anatomical marker to standardise this specific portion of the VL. Additional measurements recorded included body mass, shoulder height and body length, from which a unique quadrupedal body mass index (BMI) was calculated as described by Kirberger and Tordiffe (2016). This information, along with the respective age and sex of each animal, was recorded for each collected sample.

Immediately after each biopsy, the muscle sample was further divided into smaller pieces, rapidly frozen in liquid nitrogen and stored in a cryo-preservation tank at −200°C. The samples were transported from the clinic to the laboratory and then stored at −87°C until further analysis.

### Human participants and muscle biopsies

The skeletal muscle analyses of 13 recreational physically active (not athletes) adults (6 males and 7 females) who consented to taking part in a comparative study, were used as reference values to compare with the data from the cheetahs. Muscle biopsies of approximately 200 mg each were obtained through the suction-assisted biopsy technique using a Bergström needle from the VL muscle as previously described (Tarnopolsky et al., 2011).

### Muscle morphology and fibre type

Frozen muscle samples from the cheetahs, and from only 5 women and 5 men (because of biopsy size), were cut into serial cross-sections (10 μm thick) using a cryostat microtome (Leica CM1100, Leica Biosystems). All primary antibodies used were sourced from the Developmental Studies Hybridoma Bank (Iowa City, IA, USA) and validated for their identification of the various fibre types. Fibres expressing the type IIB isoform were identified using two specific antibodies (BF-F3 and 10F5), but none showed any positive signal in the cheetah and human fibres. All sections were fixed in chilled (−20°C) acetone for 2 min, rehydrated in phosphate-buffered saline, pH 7.40 (PBS), and blocked with 5% bovine serum albumin in PBS at room temperature for 1 h. Directly after blocking, the sections were incubated overnight at 4°C with a cocktail of monoclonal antibodies specific to MHC I (BA-D5, IgG_2b_), MHC IIA (SC-71, IgG_1_) and MHC IIX (6H1, IgM). The following day, sections were washed in PBS and incubated for 2 h with a cocktail of secondary antibodies that react with IgG_1_ (Alexa Fluor 488-conjugated goat anti-mouse; green), IgG_2b_ (AMCA-conjugated goat anti-mouse; blue) or IgM (Cy_3_-conjugated goat anti-mouse; red), all from Jackson ImmunoResearch Laboratories (West Grove, PA, USA). After washing and a brief drying cycle, the sections were mounted with MOVIOL containing antifade, allowed to set in the dark and imaged the following day using a fluorescence microscope (Nikon Eclipse 80i, Tokyo, Japan). Fibres were typed as type I, IIA and IIX. The overall number of hybrid fibres (i.e. fibres expressing more than one MHC isoform) was negligible and these were excluded from the analyses. The cross-sectional area (CSA) of the fibres was determined using the Fiji software package (Schindelin et al., 2012).

### SDS-PAGE separation of MHC isoforms

Determination of the specific MHC isoform content of the cheetah and human samples was achieved by using SDS-PAGE, as previously described by Kohn et al. (2011a). Gels were subsequently stained with Coomassie Blue, the images digitised and the densities of the bands quantified using the Un-Scan-It software package (Silk Scientific Inc., Provo, UT, USA). A rat and a lion muscle sample from previous studies were included as a reference to indicate MHC isoform migration patterns (Hohl et al., 2020; Kohn et al., 2011a).

### Maximal enzymatic activities of energy-producing pathways and antioxidant capacity

A small portion of the muscle biopsy was freeze-dried and homogenised in 0.1 mol l^−1^ potassium phosphate buffer, pH 7.40, using a sonicator. The protein content was determined using the assay developed by Bradford (1976). Maximal enzymatic activities of specific markers of the metabolic pathways were determined using fluorometric techniques, as described by Hohl et al. (2020) and Kohn et al. (2007a, 2011a). CS and 3-hydroxyacyl acetyl co-enzyme A dehydrogenase (3HAD), acting as markers of the Kreb's cycle and β-oxidation, respectively, were employed to provide a measure of oxidative metabolism. Lactate dehydrogenase (LDH), phosphofructokinase (PFK), creatine kinase (CK) and glycogen phosphorylase (GP) acted as markers of oxygen-independent metabolism, and more rapid energy production. Enzyme activity is expressed as μmol min^−1^ g^−1^ protein.

Markers of antioxidant capacity, including superoxide dismutase (SOD), catalase (CAT) and the oxygen radical absorbance capacity (ORAC) were determined as described by Hohl et al. (2020) and van Boom et al. (2022). SOD activity is expressed as U g^−1^ protein, where U was determined using xanthine oxidase, which produces oxygen radicals, whereas CAT activity is expressed as µmol H_2_O_2_ min^−1^ g^−1^ protein. ORAC values were calculated relative to a Trolox (a vitamin E analogue) standard and expressed as μmol g^−1^ protein.

### Muscle glycogen and lactate content

A separate piece of muscle was freeze-dried and the glycogen and intramuscular lactate concentrations were quantified according to previously published methods (Jensen et al., 2012). After freeze-drying, the samples were weighed and homogenised in ice-cold buffer [1:110: 50 mmol l^−1^ Hepes, pH 7.40, 150 mmol l^−1^ NaCl, 10 mmol l^−1^ Na_4_P_2_O_7_, 30 mmol l^−1^ NaF, 1 mmol l^−1^ Na_3_VO_4_, 10 mmol l^−1^ EDTA, 2.5 mmol l^−1^ benzamidine and 0.25 µl protease inhibitor cocktail (P-8340, Sigma-Aldrich-Merck) for every 1 mg dry muscle] with a Retsch MR400 mixer mill (Retsch GmbH, Haan, Germany). To determine glycogen, 60 µl of 1.8 mol l^−1^ HCl was added to 30 µl homogenate and heated at 100°C for 3 h. After hydrolysis, the samples were neutralised with 9 µl of 6 mol l^−1^ NaOH. Glucose units were measured fluorometrically according to the methods described by Passonneau and Lowry (1993). For measurement of intramuscular lactate concentration, 15 µl of 3 mol l^−1^ perchloric acid was added to 30 µl homogenate, mixed and incubated at 4°C for 15 min. The samples were neutralised with 30 µl 2 mol l^−1^ KHCO_3_ and centrifuged at 3000***g*** for 5 min at 4°C. Lactate concentration was measured fluorometrically as described by Passonneau and Lowry (1993). Glycogen and lactate were expressed as mmol kg^−1^ dry mass (dm) muscle. Corrected glycogen content, to compensate for some glycogen that was metabolised to lactate, was determined using the following equation (in mmol kg^−1^ dm): glycogen+(lactate/2) (Connett and Sahlin, 2011).

### Statistical analyses

All data are presented as means±s.d. Normality of data was checked using the Shapiro–Wilks test and using two-tailed tests. Comparisons between species and sex, assuming unequal s.d. between groups, were performed using the Brown–Forsythe and Welch one-way analysis of variance with Dunnett's *post hoc* test. Whenever two independent variables (e.g. between two sexes) were compared (e.g. CS activity between male and female cheetahs), an unpaired *t*-test with Welch's correction was used. Correlations were conducted using Pearson's *R*^2^ correlation using GraphPad Prism (v. 10.0.2). Significance was set at *P*<0.05.

## RESULTS

### Physiological parameters of cheetahs and humans

Table 1 provides the average age, body mass and BMI of cheetahs and humans, as a collective and divided into male and female. The BMI of cheetahs and humans is not comparable as the calculation of BMI for the cheetah is different to that of humans. In cheetahs, a size index calculation is based on their body length and shoulder height, as described by Kirberger and Tordiffe (2016). Male and female cheetahs were of similar age, but the females weighed less than the males, which is also reflected in their BMI. There was no statistical difference between men and women regarding age, body mass and BMI.

**Table 1.**
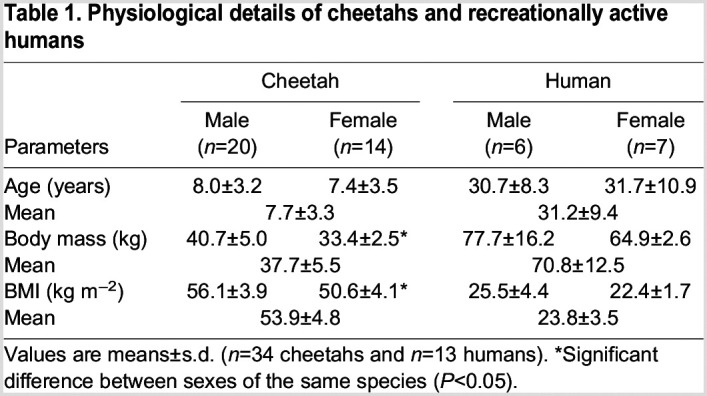
Physiological details of cheetahs and recreationally active humans

### Muscle fibre type and CSA

**Fig. 2.**
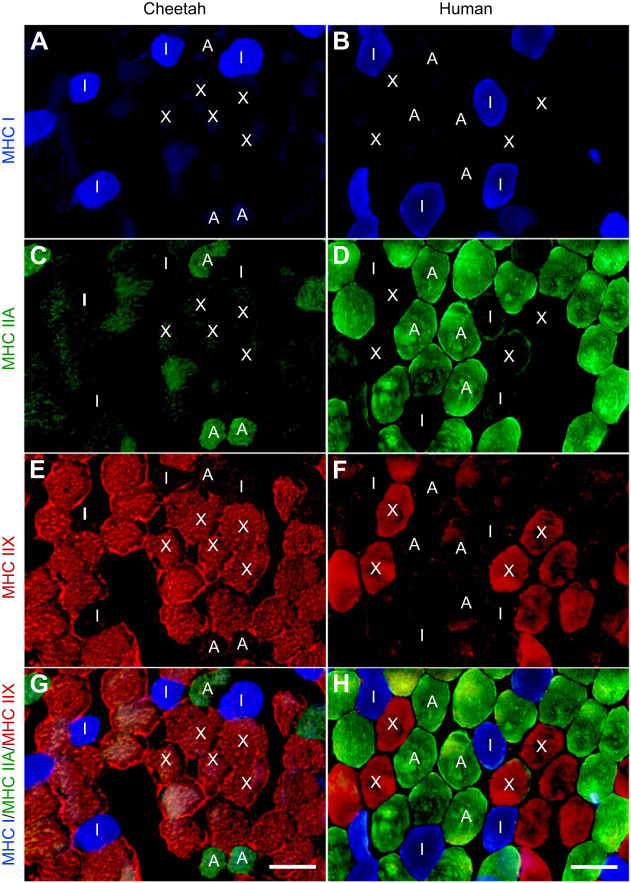
**Immunohistology of cheetah and human vastus lateralis skeletal muscle biopsies.** (A,B) Fibres which stained positively for myosin heavy chain (MHC) isoform I are shown in blue. (C,D) Fibres which stained positively for MHC IIA are shown in green. (E,F) Fibres which stained positively for MHC IIX are shown in red. (G,H) Merged images. Scale bars: 100 µm.

**Fig. 3.**
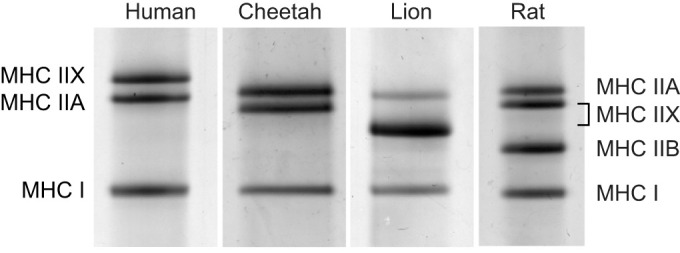
**Representative SDS-PAGE image of separated myosin heavy chain (MHC) isoforms from human and cheetah skeletal muscle.** Lion and rat skeletal muscle was included to provide reference markers to indicate the migration patterns of the various isoforms.

Immunofluorescence images that specifically identified type I, IIA and IIX fibres in cheetah and human muscles are depicted in Fig. 2. Antibodies directed against MHC I, MHC IIA and MHC IIX were able to identify type I, IIA and IIX fibres, respectively, in both species. No type IIB fibres were found, and this finding was confirmed using SDS-PAGE to separate the MHC isoforms of the cheetah and human (isoform migration patterns of four species are shown in Fig. 3).

**Fig. 4.**
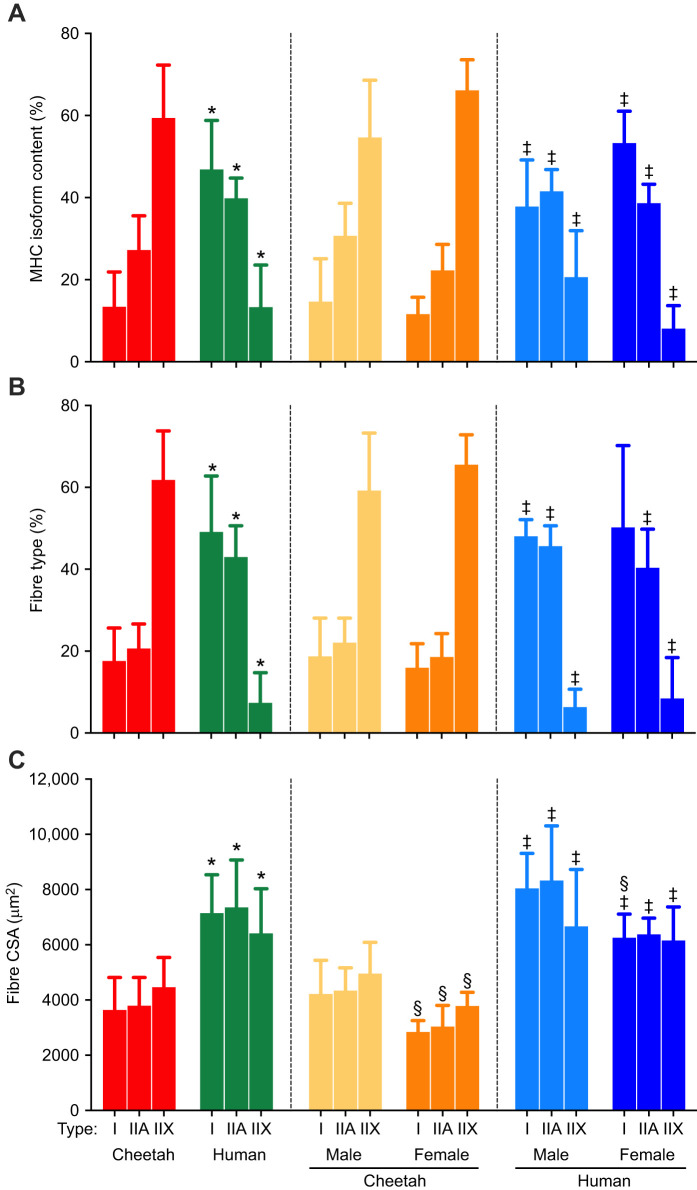
**Muscle fibre type and cross-sectional area.** (A) MHC isoform content, (B) fibre type calculated from immunohistochemistry and (C) fibre cross-sectional area (CSA) of the vastus lateralis muscle from cheetahs and humans as a species and divided into sex. For sample sizes, see Results. Data are presented as means±s.d. *Significantly different from cheetahs within the same fibre type (*P*<0.05); ^‡^significantly different from cheetahs of the same sex within the same fibre type (*P*<0.05); ^§^significantly different from males within the same species (*P*<0.05).

MHC isoform distribution and fibre types determined from immunohistochemistry are reported in Fig. 4A,B, respectively. Cheetah muscle, on average, contained predominantly type IIX fibres (∼60%), followed by type IIA (∼20%) and type I (∼20%) fibres. These proportions were also reflected in the MHC isoform distribution determined with electrophoresis in a separate piece of muscle. It appeared that the male cheetah muscles had slightly more of the MHC IIA isoform compared with the females (*P*=0.09), but overall, no difference in fibre type between male and female cheetahs was found.

Collectively, the human muscle samples contained more, and approximately equal, proportions of type I and IIA fibres compared with cheetah muscle, with type IIX fibres being the lowest (*P*<0.001) (Fig. 4B). This fibre type profile was echoed in the homogenate analyses of the MHC isoform content when a separate muscle sample was used (Fig. 4A). As for the cheetahs, there was no difference in the MHC isoform content or fibre type proportions in the biopsies from men and women (Fig. 4A,B).

The CSA of the fibres is depicted in Fig. 4C. On average, the CSA of the cheetah muscle fibres was 45% smaller than that of human fibres (3964±1022 µm^2^ versus 7205±1444 µm^2^, *P*<0.001), and differed across the three fibre types. In addition, and as expected, the fibres from males (cheetah and human) were larger than those of females (cheetah: 4505±969 µm^2^ versus 3220±501 µm^2^, *P*<0.001; human: 8131±1502 μm^2^ versus 6278±540 μm^2^, *P*<0.05). Finally, all the fibre types from female cheetahs were smaller than their male counterparts (*P*<0.05), whereas the type I fibres in humans were smaller in women than men.

### Maximal enzymatic activities of energy-producing pathways and antioxidant capacity

**Fig. 5.**
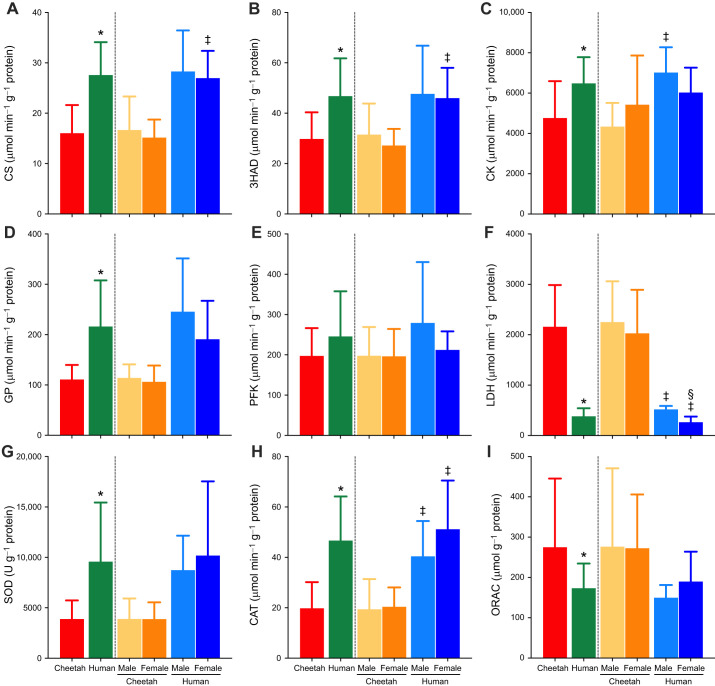
**Maximal enzyme activities of cheetah and human vastus lateralis muscle as a species and divided into sex.** (A) CS, citrate synthase; (B) 3HAD, 3-hydroxyacyl acetyl co-enzyme A dehydrogenase; (C) CK, creatine kinase; (D) GP, glycogen phosphorylase; (E) PFK, phosphofructokinase; (F) LDH, lactate dehydrogenase; (G) SOD, superoxide dismutase; (H) CAT, catalase; (I) ORAC, oxygen radical absorbance capacity. Refer to Materials and Methods for clarification on the units. Data are presented as means±s.d. (*n*=34 cheetahs and *n*=13 humans). *Significantly different from cheetahs (*P*<0.05); ^‡^significantly different from cheetahs within the same sex (*P*<0.05); ^§^significantly different from males within the same species (*P*<0.05).

Enzyme activities were measured to provide an indication of the capacity of the respective pathways to produce ATP and to combat oxidative stress (Fig. 5). Collectively, cheetah muscle had significantly lower CS and 3HAD activities compared with humans, indicating poorer oxidative capacity. When the species were divided into the two sexes, there were no statistical differences between males of the two species, but for the females, the differences were significant. CK activity was higher in human skeletal muscle compared with that in cheetahs, and only significantly higher in men than in cheetah males. Similarly, the collective GP activity was higher in humans but was not different when separated into the sexes. The rate-limiting enzyme for glycolysis, PFK, was similar between the two species and sexes. LDH activity was significantly higher in the cheetah compared with human muscle for both sexes, and this difference was also found between men and women. SOD and CAT activities were lower in cheetahs than in humans, but not different between sexes within the same species. In contrast, the ORAC assay revealed a greater overall antioxidant capacity in cheetahs compared with humans.

### Muscle glycogen, lactate and calculated glycogen content

Concentrations of glycogen, lactate and corrected glycogen in the muscles of cheetahs are shown in Table 2. The amount of glycogen varied in concentration, with some muscle samples having very low glycogen content (<10 mmol kg^−1^ dm). In contrast, the average intramuscular lactate concentrations were above normal resting muscle values (between 1 and 10 mmol kg^−1^ dm) (Karlsson et al., 1972). The inclusion of lactate in the calculation of glycogen (as glucose equivalents) provides a more realistic estimate of what the glycogen concentration may have been. By doing this, the average calculated glycogen concentration increased from 38.6 to 75.4 mmol kg^−1^ dm. There was no significant difference in muscle glycogen, lactate and corrected glycogen concentrations between male and female cheetahs.

**Table 2.**
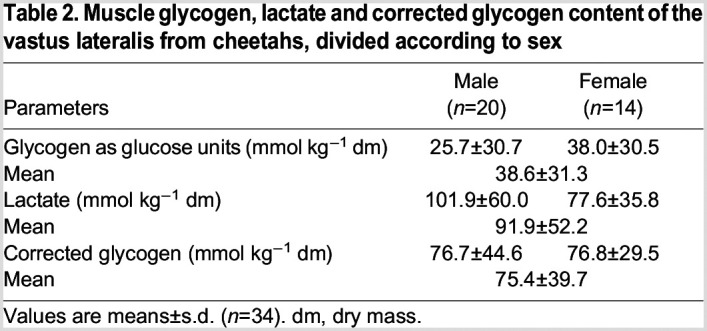
Muscle glycogen, lactate and corrected glycogen content of the vastus lateralis from cheetahs, divided according to sex

### Correlations

There was a positive correlation in the activities of 3HAD and CS in both species (cheetah: *r*=0.78, *P*<0.001; human: *r*=0.64, *P*<0.05), and combining the species (*r*=0.84, *P*<0.001). As individual species, a positive correlation was found between CK and LDH (cheetah: *r*=0.51, *P*<0.05; human: *r*=0.64, *P*<0.05), but there was no correlation when species were combined, due to clustering of data. Interestingly, LDH activity had a negative correlation with MHC I (*r*=−0.64, *P*<0.05) and positive correlation (*r*=0.57, *P*<0.05) with MHC IIA in human muscle, but no relationship in cheetahs. However, in cheetahs, MHC IIA (*r*=−0.52, *P*<0.01) and MHC IIX (*r*=0.50, *P*<0.01) correlated negatively and positively, respectively, with CK activity.

## DISCUSSION

The cheetah, being the fastest land animal known to man, remains a fascinating animal. The present study is the first to analyse a large cohort of cheetahs to better understand their muscle physiology and to subsequently determine whether sex affects their muscle fibre type and metabolism as is observed in other mammalian species. It needs to be emphasised that the animals in this study were semi-captive, and not free roaming. Therefore, the cheetahs in this study would not stalk, chase and capture their prey, and the lack of these activities could influence their muscle characteristics.

The main findings were that cheetah VL predominantly contained type IIX muscle fibres (∼60%), followed by type IIA and a small percentage of type I fibres compared with human VL (Fig. 4A,B). These proportions also conformed to the fibre type previously reported for this species (Goto et al., 2013; Williams et al., 1997). A similar pattern was observed when cheetahs were divided according to sex, with no difference found in fibre type composition between the sexes using immunohistochemistry (Fig. 4B) or MHC isoform content (Fig. 4A). The data therefore suggest that sex (i.e. male versus female) does not influence fibre type composition in humans and cheetahs, and is also supported by previous findings comparing fibre type in humans and mice, when factors such as physical activity and environment were controlled (Esbjornsson et al., 2021; O'Reilly et al., 2021).

Although Hyatt et al. (2010) reported the presence of a small number of type IIB fibres in the medial gastrocnemius of the cheetah (7–16%), no type IIB fibres were detected despite using two different antibodies against the MHC IIB isoform. Importantly, only three bands were visible on silver-stained gels (Fig. 3), documenting that only the three isoforms of MHC we detected with antibodies in the VL of cheetahs. This finding is in line with previous results where type IIB fibres were also absent in the tibialis anterior and plantaris muscles of the cheetah (Hyatt et al., 2010).

Apart from fibre type, the amount of force a fibre produces is also dependent on its CSA (Bottinelli and Reggiani, 2000). On average, there was a large difference in muscle CSA between male and female cheetahs and as well as between their respective fibre types (Fig. 4C). This finding is frequently observed in humans and animals, where males usually harbour larger fibres than their female counterparts, and is attributed to the influence of growth hormone concentrations and training status of individuals (Esbjornsson et al., 2021; Simoneau and Bouchard, 1989). Fibre CSAs have also been positively correlated with body mass, but fibre size appears to be reserved within a species (Landen et al., 2023). It is well known that in human and rodent muscles, the CSA of fibres and fibre type play significant roles in force and, ultimately, power production of a muscle (Bottinelli, 2001; Frontera and Ochala, 2015). However, Kohn and Noakes (2013) showed that the IIX fibres of lion and caracal were smaller than those of humans, but produced significantly greater specific force and three times as much power as their human equivalent. Interestingly, West et al. (2013) showed that cheetah type II single fibres produced less power than type IIX fibres obtained from rabbit psoas muscle and, thus, muscle function alone could not explain the superior running performance of the cheetah. However, these fibres were not divided into type IIA and IIX fibres, which could have caused a ‘dilution’ effect and affected the overall outcome of the power output of each fibre. The data were also obtained from one male cheetah that suffered from renal failure and was subsequently euthanised. Nevertheless, in all, the data from the present study with a larger cohort of cheetahs confirm previous findings on their muscle fibre type. Apart from the type and CSA of the fibres, the constant supply of ATP to sustain the demand of the cross-bridge cycles is also crucial to sustain optimal muscle contraction and relaxation. Therefore, the amount of substrate (i.e. glycogen) and the flux capacity of the various metabolic pathways to replenish ATP plays an important role in sustaining contraction (i.e. locomotion) and resisting fatigue (endurance capacity).

Metabolically, the VL of cheetahs had lower oxidative capacity (i.e. low 3HAD and CS activities) compared with that of their human equivalent. This agrees with a previous study and with the muscle fibre type profile of the cheetah (Williams et al., 1997). Human skeletal muscle had higher CK activity compared with cheetah muscle (*P*<0.05), with the lowest and highest activities observed in male cheetahs and men, respectively. This study is the first to have measured and compared the CK activities of cheetahs and humans. Williams et al. (1997) only measured creatine phosphate content (the substrate of CK) in the gastrocnemius of one cheetah, which amounted to 25.8 µmol g^−1^ wet mass (wm). At rest, it appears that human muscle has less creatine phosphate, ranging from 12 to ∼20 µmol g^−1^ wm (Green et al., 2007; Karlsson et al., 1970). Interestingly, Tordiffe et al. (2017) found that cheetahs excrete exceptionally large quantities of creatinine in their urine, ranging from 12.9 to 115.4 mmol l^−1^ (mean 48.64 mmol l^−1^). Humans rarely excrete more than 26.52 mmol l^−1^ (300 mg dl^−1^) (Barr et al., 2005). This seems to indicate that cheetahs produce far more creatine endogenously than humans. Because of the low sample mass collected from both species, creatine phosphate could not be measured in the present study.

GP activity, the enzyme that mobilises glucose 1-phosphate from glycogen, was approximately two times lower in cheetah muscle compared with human muscle (Fig. 5D), with both sexes have similar activity. GP protein content was not measured and would have shed light on the differences observed between cheetah and human muscle. Nevertheless, the significance of this finding could indicate that there is a limitation on the cheetah to mobilise their glycogen stores.

In contrast, LDH activity was six times higher in cheetah muscle compared with their human counterparts, and shared similarity between the sexes for the same species. LDH converts pyruvate to lactate, which is shuttled from the contracting muscle fibres to the blood via monocarboxylate transporters (Brooks et al., 2023). The same pattern of high LDH and lower GP and CK activities was found in the muscle of a caracal and two female lions (Kohn et al., 2011a). As one glucose molecule produces two pyruvates, it is still not yet clear whether a limitation in substrate availability occurs. What is important to note is that the prey of cheetahs and other felids, usually smaller antelope, also have high LDH activity. Additionally, these animals appear to have an added advantage of having high mitochondrial numbers, and thus high CS activity, providing a greater resistance to fatigue (Curry et al., 2012; Kohn, 2014; Kohn et al., 2011b). Indeed, Hetem et al. (2013) indicated that cheetahs do not abandon their hunts because of overheating, but rather from a yet unidentified cause, which could be metabolic. In theory, this may indicate that during an all-out sprint (i.e. chasing prey), cheetahs merely become metabolite depleted in the form of low muscle glycogen and creatine phosphate concentrations, which induces muscle fatigue. Whether cheetahs can deplete their muscle glycogen within 30 to 45 s is yet to be determined.

The glycogen content of the cheetah muscle varied significantly, as reflected in the large standard deviations, but this was not surprising as it was previously shown in human muscle (Jensen et al., 2012). Similarly, intramuscular lactate concentrations were also elevated in some cheetah biopsies. Indeed, muscle lactate was notably elevated in the cheetah muscle compared with previous literature in humans. Concentrations between 1 and 10 mmol lactate kg^−1^ dm muscle have been deemed normal at rest in humans (Edge et al., 2013; Karlsson et al., 1972). Although only measured in one cheetah at rest, the lactate concentration within the muscles also fell within this range (Williams et al., 1997). Including lactate in the calculations for glycogen resulted in higher corrected muscle glycogen concentration, and conformed to what was reported by Williams et al. (1997), but was still lower than what is generally reported for humans and other species (Essén-Gustavsson and Rehbinder, 1985; Guy and Snow, 1981; Kohn et al., 2023; Webster et al., 2016). The most logical explanation for the low glycogen content is that the cheetahs in the present study were semi-captive, in that they were not accustomed to human presence and could therefore have been stressed. In addition, the darting resulted in some cheetahs briefly running wild (flight response) and could have significantly impacted their glycogen content. However, glycogen content is not limiting to anaerobic capacity in humans, where approximately 25% is degraded and the accumulation of lactate and other metabolites has been associated with causing fatigue. Therefore, and speculatively, the higher glycogen content in humans may have a beneficial effect on resistance to fatigue, but cheetahs may not have the same need for high muscle glycogen content (Bergstrom et al., 1967). Moreover, glycogen binds water and will increase body mass. Cheetahs may, thus, inherently store low quantities of glycogen with a reduced ability to use it for ATP production during a hunt (average between 20 and 45 s sprint), which could result from their low GP activity (Hetem et al., 2013). Therefore, even though the glycogen content was also found to be low in the study by Williams et al. (1997), the true resting glycogen content of the cheetah remains to be determined. In all, there was no difference between the sexes.

Although a few correlations were found between fibre type and some of the enzymes, their biological relevance is unclear. The positive relationship between 3HAD and CS, as individual species or combined, was also not a surprise as these two enzymes are found in the mitochondria.

It is well known that reactive oxygen species can cause muscle fatigue to prematurely set in. What was noteworthy was the low SOD and CAT activities measured in the cheetah muscle compared with human muscle, although total antioxidant capacity (ORAC) was higher in the cheetahs (Fig. 5). The total antioxidant capacity of tissues relies on the cumulative effect of all the known and unknown antioxidants (Ghiselli et al., 2000). Therefore, the other antioxidant enzymes (e.g. glutathione peroxidase, peroxiredoxins) and proteins acting as antioxidants can result in the finding that cheetah muscle overall has a greater antioxidant capacity. The breakdown of high levels of intracellular AMP from muscle contraction results in significant free radical production, which requires neutralisation. The high rate of ATP utilisation by the muscles of the cheetah during an all-out sprint would generate significant quantities of AMP and, hence, free radicals, and would thus be countered by the high total antioxidant capacity. It would, therefore, be valuable for future studies to investigate the activities of other antioxidant enzymes (such as glutathione peroxidase) in order to better understand their total antioxidant capacity.

### Conclusion

This is the first study to compare the muscle characteristics of a large cohort of cheetahs with those of recreationally active humans. Overall, in relation to human muscle, cheetah muscle had poor oxidative capacity (and may be less resistant to fatigue) due to the predominance in type IIX fibres and lower activities of their mitochondrial enzymes, CS and 3HAD. Male cheetahs had larger fibres than their female counterparts, conforming to sex differences frequently observed between men and women. Interestingly, cheetahs had lower CK and GP activity compared with humans, which may indicate a limitation in substrate availability or utilisation of creatine phosphate and glycogen, which was highlighted by the lower concentration of glycogen and corrected glycogen compared with previous human studies. The higher LDH activity of the cheetah may be due to the reduction in the activity of other enzymes in the respective pathways. Cheetahs also exhibited higher overall antioxidant capacity compared with humans. The findings of the current study provide valuable information on the skeletal muscle of the cheetah and help explain the high-speed characteristics of this endangered felid.
